# Residual Microscopic Peritoneal Metastases after Macroscopic Complete Cytoreductive Surgery for Advanced High-Grade Serous Ovarian Carcinoma: A Target for Folate Receptor Targeted Photodynamic Therapy?

**DOI:** 10.3390/ph15081034

**Published:** 2022-08-22

**Authors:** Morgane Moinard, Jeremy Augustin, Marine Carrier, Elisabeth Da Maïa, Alix Penel, Jérémie Belghiti, Maryam Nikpayam, Clémentine Gonthier, Geoffroy Canlorbe, Samir Acherar, Nadira Delhem, Céline Frochot, Catherine Uzan, Henri Azaïs

**Affiliations:** 1 Laboratoire des Réactions et Génie des Procédés (LRGP), CNRS-Université de Lorraine, 1 rue Grandville, 54000 Nancy, France; 2 AP-HP, Pitié-Salpêtrière Hospital, Department of Pathology, 75013 Paris, France; 3 Department of Gynecological and Breast Surgery and Oncology, Assistance Publique-Hôpitaux de Paris (AP-HP), Pitié-Salpêtrière University Hospital, 75013 Paris, France; 4 AP-HP, Pitié-Salpêtrière Hospital, Centre de Pharmaco-épidémiologie de l’APHP (CEPHEPI), 75013 Paris, France; 5 Cancer Biology and Therapeutics, Centre de Recherche Saint-Antoine (CRSA), Sorbonne University, INSERM UMR_S_938, 75020 Paris, France; 6 Institut Universitaire de Cancérologie (IUC), Sorbonne University, 75013 Paris, France; 7 Laboratoire de Chimie-Physique Macromoléculaire (LCPM), CNRS-Université de Lorraine, 54000 Nancy, France; 8 INSERM, CHU-Lille, U1189-ONCO-THAI—Assisted Laser Therapy and Immunotherapy for Oncology, University of Lille, 59000 Lille, France

**Keywords:** epithelial ovarian cancer, peritoneal carcinomatosis, folate receptor, photodynamic therapy, cytoreductive surgery, gynecologic oncology

## Abstract

Despite conventional treatment combining complete macroscopic cytoreductive surgery (CRS) and systemic chemotherapy, residual microscopic peritoneal metastases (mPM) may persist as the cause of peritoneal recurrence in 60% of patients. Therefore, there is a real need to specifically target these mPM to definitively eradicate any traces of the disease and improve patient survival. Therapeutic targeting method, such as photodynamic therapy, would be a promising method for such a purpose. Folate receptor alpha (FRα), as it is specifically overexpressed by cancer cells from various origins, including ovarian cancer cells, is a good target to address photosensitizing molecules. The aim of this study was to determine FRα expression by residual mPM after complete macroscopic CRS in patients with advanced high-grade serous ovarian cancer (HGSOC). A prospective study conducted between 1 June 2018 and 10 July 2019 in a single referent center accredited by the European Society of Gynecological Oncology for advanced EOC surgical management. Consecutive patients presenting with advanced HGSOC and eligible for complete macroscopic CRS were included. Up to 13 peritoneal biopsies were taken from macroscopically healthy peritoneum at the end of CRS and examined for the presence of mPM. In case of detection of mPM, a systematic search for RFα expression by immunohistochemistry was performed. Twenty-six patients were included and 26.9% presented mPM. In the subgroup of patients with mPM, FRα expression was positive on diagnostic biopsy before neoadjuvant chemotherapy for 67% of patients, on macroscopic peritoneal metastases for 86% of patients, and on mPM for 75% of patients. In the subgroup of patients with no mPM, FRα expression was found on diagnostic biopsy before neoadjuvant chemotherapy in 29% of patients and on macroscopic peritoneal metastases in 78% of patients. FRα is well expressed by patients with or without mPM after complete macroscopic CRS in patients with advanced HGSOC. In addition to conventional cytoreductive surgery, the use of a therapeutic targeting method, such as photodynamic therapy, by addressing photosensitizing molecules that specifically target FRα may be studied.

## 1. Introduction

Epithelial ovarian cancer (EOC) is most often diagnosed at an advanced stage, when peritoneal metastases are already present within the abdominal cavity. Despite a treatment combining complete macroscopic cytoreductive surgery (CRS) (which is the surgical removal of all peritoneal lesions suspected of cancer and visible to the surgeon) with platinum-based chemotherapy [1,2,3], 60% of patients suffering from EOC will present peritoneal recurrence after primary clinical remission [4,5].

Despite improvements in surgical skills and treatment strategies, microscopic clusters of cancer cells that have not been eradicated by either surgery or chemotherapy persist after CRS and thus may lead to recurrence [6]. Thus, particular attention needs to be paid to the improvement of locoregional treatment strategies in addition to conventional surgery, especially given that peritoneal progression is responsible for morbidity and complications leading to death in numerous cases. The absence of macroscopic residual disease after CRS has been shown to improve prognosis with a high level of evidence [7,8,9,10,11,12,13,14], and this observation could be extrapolated to the treatment of microscopic peritoneal involvement, even in the absence of dedicated clinical study.

We recently published a study suggesting that the presence of residual microscopic PM (mPM) was almost constant [15], which is in line with what was supposed. Techniques that could further decrease the peritoneal tumor burden in addition to conventional surgery include intraperitoneal chemotherapy (with or without hyperthermia), fluorescence-guided surgery, and photodynamic therapy (PDT). Photodynamic therapy is an effective technique that has already been applied in other medical indications, widely in dermatology and in oncological indications [16]. After administration of a photosensitizer (PS) that accumulates in cancer cells, illumination with a light of adequate wavelength induces a photochemical reaction leading to a cytotoxic phenomenon. Its ability to treat superficial cancer lesions over a large area makes it an excellent candidate for the destruction of microscopic peritoneal residual disease on the peritoneum. To enable intraperitoneal PDT of EOC PM, a precise targeting of lesion is mandatory. The development of intraperitoneal PDT has been limited because of poor tolerance due to the lack of specificity of PS and the proximity of intraperitoneal organs. First-generation photosensitizers (porfimer sodium and dihematoporphyrin ethers) are the only PS that have been evaluated in intraperitoneal indications in phase I and II clinical trials [17,18,19]. The authors in these studies report severe morbidity such as digestive perforations, capillary leak syndrome, and anastomotic leakages, with no benefit to recurrence-free or overall survival. This limited therapeutic window [18] has been attributed to a narrow differential of drug selectivity between the tumor and normal tissue in the peritoneal cavity [20]. Molecularly targeted PS then have a strong clinical potential and are needed to improve the therapeutic index of intraperitoneal PDT [21]. The folate receptor alpha (FRα) is a target that would allow for addressing a photosensitizing molecule with excellent specificity for the complementary treatment of mPM of ovarian origin.

The objective of this study was to determine FRα expression by residual mPM after complete macroscopic CRS in patients with advanced high-grade serous ovarian cancer (HGSOC).

## 2. Results

### 2.1. Population Characteristics

Twenty-six patients with advanced HGSOC were included after complete macroscopic CRS. Among them, seven (26.9%) presented with mPM. The characteristics of the entire population, and those of the two subgroups (without or with mPM), are presented in Table 1.

The mean age of the patients at the time of diagnosis was 65.3 years +/- 11.1.

All the patients received chemotherapy with carboplatin and paclitaxel. Chemotherapy was administered in a neoadjuvant setting (NACT) for 23 patients (88.5%). The median follow-up time was 492 [262–862] days. During this period, eight (30.8%) recurrences occurred with a median follow-up time of 356 [213–862] days, six (31.6%) in the group without mPM and two (28.6%) in the group with mPM. Three patients died during the study period.

There was no statistical difference between the two groups regarding Peritoneal Carcinomatosis Index (PCI) at diagnosis (*p* = 0.16) or at the time of CRS (*p* = 0.40), the Fagotti score at diagnosis (*p* = 0.26) or at the time of CRS (*p* = 0.97), or the CA 125 levels at diagnosis (*p* = 0.57) or at the time of CRS (*p* = 0.30), although these parameters were still higher at diagnosis for the patients with identified mPM.

The number of courses of NACT did not differ in patients with or without mPM (*p* = 0.84).

### 2.2. Analysis of Peritoneal Biopsies in Macroscopically Healthy Peritoneum

A median of 7 [3,4,5,6,7,8,9,10,11,12,13] biopsies were taken per patient with no difference between the two groups: 6 [3,4,5,6,7,8,9,10,11,12] for patients with mPM and 7 [3,4,5,6,7,8,9,10,11,12,13] for patients without (*p* = 0.41).

Microscopic PM were located in the Morrison space (*n* = 2), small omentum (*n* = 2), right diaphragmatic peritoneum (*n* = 2), pelvic peritoneum (*n* = 1), parieto-colic gutter (*n* = 1), mesorectal peritoneum (*n* = 1), mesenteric peritoneum, and prevesical peritoneum (*n* = 1).

### 2.3. Tissue Expression of the Folate Receptor α Isoform (FRα)

**Figure 1:**
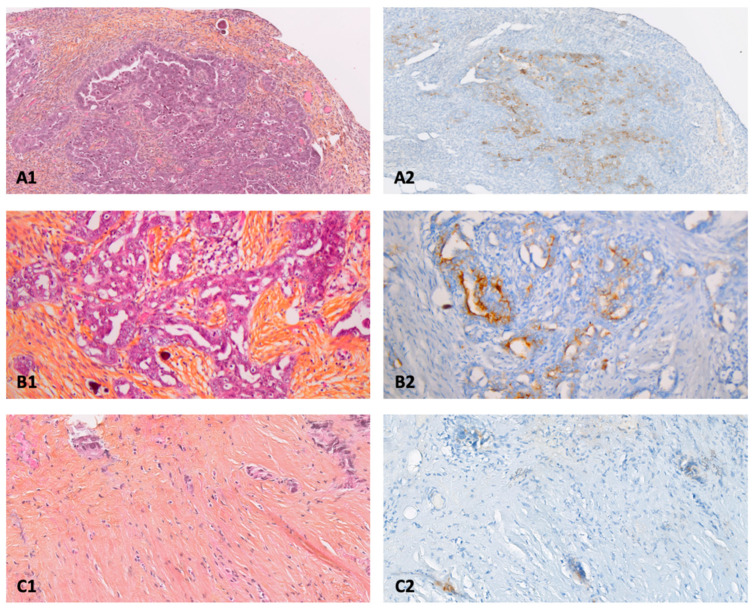
Anatomopathological examination of primary and peritoneal lesions of high-grade serous ovarian carcinoma (HES: Hematein, Eosin, Saffron), and expression of folate receptor alpha (FRα) by immunohistochemistry. (**A1**): primary cancer × 10/(**A2**): FRα (75%; 3), (**B1**): macroscopic peritoneal metastase × 20/(**B2**): FRα (30%; 3), (**C1**): microscopic peritoneal metastases × 10/(**C2**): FRα (30%; 2), FRα (percentage of FRα positive tumor cells; staining intensity).

The expression of FRα was studied for all patients on the following specimens (Figure 1):

- Diagnostic biopsy sampled during initial exploratory laparoscopy if available;
- Macroscopic PM sampled during CRS;
- mPM if identified.

In the subgroup of patients with mPM, an FRα expression study could not be performed in three cases due to tumor sizes that were too small for slide reading. Immunostaining could not be performed on the diagnostic biopsy in one case, as exploratory laparoscopy was performed in another center.

In the subgroup of patients with mPM, *FRα* expression was positive on diagnostic biopsy before NACT for 67% of patients, on macroscopic PM for 86% of patients, and on mPM for 75% of patients (Table 2).

Nineteen of the twenty-six patients in the study had no mPM. Of these, 84.2% received NACT (*n* = 16).

In the subgroup of patients with no mPM, FRα expression was found on diagnostic biopsy before NACT in 29% of patients and on macroscopic PM in 78% of patients. A significant number of diagnostic biopsies on exploratory laparoscopy (*n* = 12) could not be analyzed because of too small samples or when the exploratory laparoscopy was performed in another center (Table 3).

## 3. Discussion

Our previous results have demonstrated the presence of residual mPM after complete CRS. It was shown that it was highly probable (98.14% of cases) that patients suffering from advanced stage HGSOC present microscopic tumor residues after the completion of macroscopic complete CRS. Furthermore, the high rate of peritoneal microscopic spread in EOC was evidenced by the presence of mPM in more than 69 residual sites in 95% of patients [15]. Furthermore, 60% of women with EOC relapsed after conventional treatment, which combines complete macroscopic CRS with platinum-based chemotherapy [4]. One hypothesis was that this high recurrence rate was strongly related to the presence of mPM at the end of CRS. This hypothesis could therefore be supported by our previous study. Consequently, it is important to specifically target these mPM after macroscopic CRS, in order to limit their occurrence and thus increase the survival of the patients after treatment. 

**Figure 2:**
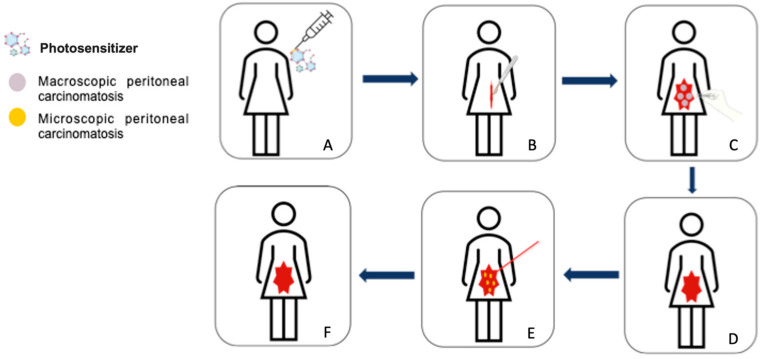
Intraperitoneal photodynamic therapy protocol for peritoneal metastases of advanced ovarian cancer. (**A**) Administration of the photosensitizer, (**B**) open approach (laparotomy) to perform macroscopic complete cytoreductive surgery, (**C**) cytoreductive surgery (hysterectomy, bilateral adnexectomy, omentectomy, appendectomy +/- pelvic and para-aortic lymphadenectomies, removal of all visible peritoneal metastases), (**D**) end of the cytoreductive surgery, (**E**) illumination of the peritoneal cavity to treat by photodynamic therapy microscopic peritoneal metastases, (**F**) end of the procedure.

Photodynamic therapy (PDT) could be a relevant alternative for the treatment of these microscopic and non-visible intraperitoneal lesions to improve the quality of cytoreduction. PDT is a technique that relies primarily on the activation of a photoactivable molecule (PS) by a light source at an appropriate wavelength. After administration of the PS to the patient, the PS accumulates in the diseased tissue. Upon illumination, the PS changes from its ground state to a singlet excited state. A part of the singlet excited states goes to the triplet excited state by an inter-system crossing process. The triplet excited states can transfer its energy to the molecular oxygen present in the medium or perform electron or proton transfer to biomolecules, to form reactive oxygen species (ROS) such as singlet oxygen ^1^O_2_. These ROS are toxic for cancer cells, leading to their destruction [22] (Figure 2). However, several drawbacks have been associated with the use of PDT, in particular the lack of specificity of PS that also accumulates in healthy tissues, leading to their damage and important side effects such as a strong photosensitivity [23].

In view of this lack of specificity, therapeutic targeting is essential. Among the different strategies, one of them is based on targeting cancer cells by addressing molecules, which bind specifically to membrane receptors overexpressed on the surface of these cancer cells. Of these receptors, the folate receptor represents a promising target. The alpha isoform of the folate receptor (FRα) is a well-known target that is overexpressed in many types of cancer (lung, breast, kidney, brain, endometrial, colon, etc.), and especially epithelial ovarian cancers [24], while having a limited expression in healthy cells or tissues. Thus, folic acid, being a ligand with a high affinity for these receptors, can be used as a targeting molecule, able to recognize and bind specifically to FRα receptors. Drugs that have been linked to folic acid for tumor-selective drug delivery include protein toxins, chemotherapeutic agents, gene therapy vectors, oligonucleotides, magnetic resonance imaging (MRI) contrast agents, liposomes with entrapped drugs, immunotherapeutic agents, enzyme constructs for prodrug therapy [25], but also PS for the development of targeted photodynamic therapy [26]. The coupling of a PS to folic acid would allow folate-conjugated drugs to bind specifically to FRα and thus enhance the internalization of the PS inside cancer cells via an endocytosis process mediated by FRα receptors.

The objective of our study was then to determine the expression of FRα by residual mPM after complete macroscopic CRS, in patients with HGSOC. According to our findings, FRα expression on macroscopic PM at CRS was positive in 85% and 67% of patients with and without mPM, respectively. Finally, FRα was also expressed by 75% of patients on the mPM. These results highlight the high expression level of FRα by PM in patients suffering from HGSOC and reinforce the idea of targeting the folate receptor by using a targeted therapy such as PDT via the use of a PS coupled with folic acid.

Over the last few years, particular attention has been focused on the synthesis of photosensitizers coupled to folic acid, with the aim of improving its selectivity for diseased tissues by limiting its accumulation in healthy tissues, improving its phototoxicity while avoiding cytotoxicity in the dark.

Nanoparticle delivery systems have shown a growing interest in recent years, because of their ability to increase the efficacy and the specific delivery of the encapsulated photosensitizer at the tumor site, due to the enhanced permeability and retention (EPR) effect, which constitutes a first level of selectivity. A dozen studies on the treatment of ovarian cancer cells by PDT, via the use of a PS-folic acid addressing system have been identified.

Karges et al. recently presented a drug delivery system based on mesoporous silica nanoparticles (MSN) conjugated to folic acid, with a size of about 200 nm. This newly described system was based on the combination of the EPR effect with the covalently attached folic acid moiety on the MSN, which represents two levels of selectivity. [Ru(bphen)_2_(4,4′-dimethyl-2,2′-bipyridine)]-[PF_6_]_2_ (bphen = 4,7-diphenyl-1,10-phenantroline) derivatives were chosen as PS and have been covalently attached to the MSN. The potential as PS for PDT was confirmed by their ability to generate ^1^O_2_ in aqueous solution upon illumination. Furthermore, they were able to put into evidence no cytotoxic effect in the dark in both normal expression of folate receptor (noncancerous human normal lung fibroblast (MRC5)) cells and folate receptor overexpressing cancerous human ovarian carcinoma (A2780) cells. Furthermore, the importance of the functionalization with folic acid was demonstrated by the fact that non-formulated nanoparticles demonstrated phototoxic effect for both cell lines upon illumination at 480 nm (8.7 J/cm²) or 540 nm (9.5 J/cm²), while functionalized MSN were only phototoxic for FRα overexpressing ovarian cancer cell lines [27].

Potara et al. recently synthetized a nanotheranostic delivery platform to treat ovarian cancer. They successfully synthetized Plu-IR780-chit folic acid nanocomposites of about 30 nm, by encapsulating IR780 NIR dye into polymeric Pluronic-F127-chitosan nanoformulation followed by functionalization with folic acid. It turned out that ^1^O_2_ quantum yield was 11% in ethanol comparable to that of free IR780. By achieving biological tests on folate receptor expressing OVCAR-3 cells, strongly reduced cytotoxicity was observed. The specific targeting of the folate receptor was highlighted by using NIR fluorescence microscopy. The team also showed that this new folic acid-functionalized system was highly internalized by a folate receptor (NIH:OVCAR-3) overexpressing ovarian cancer cells, while it was only weakly internalized by FR-negative ovarian cancer cells (A2780-cis). This proves that internalization is specifically mediated by folate receptors, reconfirming the importance of folic acid as an addressing molecule. Consistent with the latter results, functionalized nanocarriers showed a better phototherapeutic effect on NIH:OVCAR-3 cells than free IR780, under 785 nm laser illumination (160 mW, 330 s). Moreover, this system is efficient for NIRF imaging and PTT. Thus, this new folic acid-targeted phototherapeutic nanoplatform could considerably decrease the cytotoxicity of free IR780, while having a better hydrophilicity, cellular uptake and better photodynamic therapy ability [28]^.^

With the same purpose of obtaining theranostic nanoparticles, upconversion nanoparticles (UCNPs) with a size of 223.6 ± 68.86 nm were synthetized by the team of H. Wang et al. coupled to Rose Bengal (RB). Their novel nanosystem (FURH-PHF-NPs) was based on a lipid shell containing UCNPs-RB, 10-hydroxycamptothecin (HCPT), modified on the surface by folic acid as targeting molecule, and perfluorohexane (PHF) core. High reactive oxygen species formation by the FURH-PHF-NPs after illumination at 980 nm (0.2 W/cm²) was first put into evidence to release PFH, UCNPs-RB and HCPT by in vitro evaluation in SKOV3 ovarian cancer cells. The authors proved that illumination and low-intensity focused ultrasound LIFU, FURH-PFH-NP presented a high phototoxicity, better than when they were irradiated only with laser alone. The specific targeting of cancer cells and the enhanced accumulation of the NPs in the tumor region followed by their internalization could also be demonstrated. These results again demonstrate the role of folic acid as an efficient addressing molecule in receptor-mediated active targeting. In vivo studies were conducted in nude mice bearing SKOV3 xenografts. The tumor volume evaluation within 28 days revealed that without injection of FA-functionalized nanoparticles, the tumor volume kept increasing; for FURH-PFH-NPs combined with laser only, the volume was maintained while it was significantly decreased by adding LIFU. Therefore, this new nanosystem had a stronger antitumor effect when combined with laser and LIFU. These results make the folic acid-functionalized NPs + laser + LIFU system a promising therapy for the treatment of ovarian cancer [29].

Another example of the use of PDT via folic acid-coupled PSs to specifically target ovarian cancer cells was described by Bazylińska et al. They combined the use of PDT with electroporation, in order to increase the PSs cellular uptake and the photodynamic activity. They synthetized nanocarriers with a hydrodynamic diameter of 200 ± 7 nm, coated with folic acid-functionalized poly(lactide-co-glycolide) (PLGA) and which encapsulated Verteporfin with a low quantity of cisplatin. The cellular internalization and anticancer activity were studied in vitro on human ovarian cancer cell SKOV-3 and on hamster ovarian fibroblastoid CHO-K1 cell lines as control. By flow cytometry, it could be revealed that a more significant cellular internalization was obtained in the case of nanoparticles functionalized with folic acid. These results thus show the capacity of these nanocarriers to specifically target the folate receptors present on the surface of SKOV-3 cells. The effectiveness of using electroporation in combination with PDT was demonstrated by confocal microscopy studies where the uptake of FA-PLGA-coated nanoparticles was stronger than in the case of illumination alone. Furthermore, the phototoxicity was demonstrated by studying the cell viability of SKOV-3 cells after 24 h of incubation with the nanocarriers and 10 min of exposition to EP-PDT, with an illumination between 630–680 nm (10 J/cm²). They could observe a decrease in SKOV-3 cell viability of 60% to 80% at high concentration and incubation time, whereas a low decrease was observed in the case of CHO-K1 [30].

Li et al. described in 2018 the synthesis of a new folate-conjugated polymeric micelle for the target of intraperitoneal ovarian cancer cells. The polymer poly(ethylene glycol) (PEG)-poly(lactic acid) (PLA) was used to form micelles and was then conjugated to folic acid to represent a drug delivery and targeting system for the encapsulated hypocrellin B. The resulting HB/folic acid-PEG-PLA micelles have a mean diameter of about 173.8 ± 3.2 nm. They used SKOV-3 cells for FRα highly expressing cell line and two other cell lines HO8910 and A2780, which express it weakly. The cellular internalization of HB/FA-PEG-PLA micelles was strongly increased in SKOV3 cells and was stronger than in the case of non-folic acid functionalized micelles, demonstrating that folate receptors essentially mediate the uptake of the micelles. Furthermore, free HB and non-folic acid-functionalized micelles showed weaker phototoxic effect upon illumination at 650 nm (3 J/cm²), which reinforces the importance of folic acid-targeting strategy. No toxicity in the dark was observed. In vivo studies were conducted in SKOV3 tumor-bearing mice. They found that the functionalization of micelles with folic acid showed stronger accumulation of HB in tumor tissue. HB/FA-PEG-PLA micelles are once more the proof that a folic acid-mediated delivery system strongly enhances the antitumor effect of a non-functionalized system, while improving the pharmacokinetic and biodistribution characteristics in this case [31].

The last example that can be found in the literature using folic acid-functionalized nanoparticles for the treatment of ovarian cancer by PDT is the work of Doshi et al., published in 2015. In this study, they used the promising characteristics of conducting polymers to propose a new blended poly[2-methoxy-5-(2-ethylhexyl-oxy)-*p*-phenylenevinylene] (MEH-PPV)/polystyrene graft ethylene oxide functionalized with carboxylic acid (PS-PEG-COOH) nanoparticles conjugated with folic acid (FNPs) for targeting treatment of ovarian cancer. No photosensitizer was doped on these nanoparticles because the blended copolymer MEH-PPV is able to generate ^1^O_2_ by itself. Synthetized by self-aggregation, these functionalized nanoparticles have a size of 68.80 ± 7.30 nm. In vitro studies were conducted with OVCAR3 ovarian cell lines as the FRα-positive cell line, MIA PaCa-2 and A549 as FRα-negative cell lines and TE 71 as control cell line. Strong uptake of FNPs was revealed by a strong fluorescence in OVCAR3 cell lines, whereas no uptake was detectable in the three other cell lines. Flow cytometry revealed that 85% of the overexpressing folate receptor OVCAR3 cell lines internalized FNPs, against 0% for the other. No cytotoxicity was observed in the dark. This high uptake of FNPs therefore led to a near complete cell mortality of OVCAR3 cells after PDT treatment (visible light with UV filter, 180 J/cm²), whereas cell viability for TE71, Mia PaCa-2 and A549 cell lines were nearly 100%. These results strongly demonstrate the receptor-mediated uptake of the FNPs, due to the functionalization of the nanoparticles with folic acid [32].

To the best of our knowledge, the first photosensitizer coupled with folic acid for the treatment of peritoneal metastases of ovarian cancer by PDT, without the use of any nanosystem, is our work published in 2016. We developed a carboxylic tetraphenyl porphyrin coupled to folic acid via a PEG-spacer. Through in vivo studies with NuTu-19 epithelial ovarian cancer in female Fischer 344 rats, we were able to demonstrate the specific internalization of folic acid-conjugated PS in tumor tissue; the mean tumor-to-normal tissue was 9.6. These results thus demonstrate the specific internalization of folic acid-functionalized PS through the overexpression of folate receptors.

In vitro experiments in NuTu-19 ovarian cells allowed us to observe fluorescence after incorporation of the functionalized PS. The phototoxic ability of the new FA-conjugated PS was studied in vitro on the SKOV-3 cell line, by using an illumination (670 nm, 4.3 J/cm²). No dark cytotoxicity was observed. Ovarian tumor cells were completely killed 24 h post-treatment, while PS alone or light alone showed no cell mortality. The incorporation of PS through folic acid targeting is again demonstrated, thus reinforcing the interest for this new approach of folic acid targeting of mPM [33].

Our team published in 2020 biological results about the use of the new patented folate receptor-targeted PS (pyropheophorbide *a*—polyethylene glycol—folic acid (PS) (patent WO201901639711)) on peritoneal ovarian cancer. For in vitro studies, two ovarian cancer cell lines were used, SKOV3 and OVCAR3. Calculations of viability percentage for OVCAR3 and SKOV3 showed that when cell lines were subjected to PDT by combining the use of PS and illumination (668 nm, 1 mW/cm²), cell mortality was total in both cell lines 48 h post-illumination. In contrary, when they were only irradiated by light or only in the presence of the folic acid-targeted photosensitizer, no cell mortality was observed, therefore concluding that it was not phototoxic in the dark. The cell mortality was also put into evidence by observation of the shape of the cells after PDT treatment. They could observe a narrowed size and a detachment of cells to each other. These results confirm the efficacy of this new PS for treatment of peritoneal carcinomatosis by folate receptor mediated PDT [34].

All these data confirm the good efficacy of the use of PDT for the treatment of EOC in preclinical studies, using PS or nanoparticle coupled with folic acid for specific targeting toward the folate receptor. Our results demonstrate the overexpression of FRα by mPM. Combining PDT to eradicate all microscopic residues that still persist after maximal CRS could significantly decrease the recurrence rate of patients and thus increase their survival.

In addition, almost all PSs described previously by the different studies mentioned emit fluorescence under illumination. As these PSs are specifically delivered to the tumor of interest; this important physicochemical property would allow for the combination of PDT with fluorescence-guided surgery.

## 4. Methods

### 4.1. Design

This was a prospective single-center study conducted between 1 June 2018 and 10 July 2019 at the Department of Gynecological and Breast Surgery and Oncology, Pitié-Salpêtrière University Hospital (Paris, France). The objective of this ancillary analysis of the microPCI study (ClinicalTrials.gov Identifier: NCT03754569) was to determine the tissue expression of FRα by residual mPM after complete macroscopic CRS in patients with advanced high-grade serous ovarian cancer (HGSOC).

### 4.2. Funding

The study was sponsored by Assistance Publique—Hôpitaux de Paris (Délégation à la Recherche Clinique et à l’Innovation). Award number: F101H1. Recipient: Henri Azaïs, M.D., Ph.D.

The funding made it possible to purchase the consumables necessary for the analysis of the biopsies as well as to finance the administrative costs incurred to submit the project and monitor it (recruitment of staff for data collection, etc.).

### 4.3. Patients

All women over 18 years old who presented advanced-stage HGSOC at our center during the study period were invited to participate if complete macroscopic CRS could be performed at primary or interval CRS. Advanced stage was defined by stage IIB-IV of the 2014 classification of the International Federation of Gynecology and Obstetrics (FIGO) [35].

The patients were treated in accordance with international recommendations after systematic pre-therapeutic validation during a multidisciplinary consultation. Our department of surgery is certified by the European Society of Gynecologic Oncology (ESGO) for the management of advanced stage EOC. An exploratory laparoscopy was performed to assess the resectability of the lesions. The Peritoneal Carcinomatosis Index (PCI) [36] and the laparoscopic Fagotti score [37] were used to quantify macroscopic peritoneal spread. Complete macroscopic CRS was performed by laparotomy at the same time as, or a few days after, laparoscopy. Macroscopic complete CRS included at least peritoneal cytology, total hysterectomy, bilateral salpingo-oophorectomy, infragastric omentectomy, appendectomy, and other surgical procedures allowing for the removal of all visible suspicious peritoneal lesions. Pelvic and para-aortic lymphadenectomy were performed according to the recommendations [38] and results of the LION study [39]. When there was no suspicion of radiological lymph node involvement during initial management or at palpation during surgery, we did not perform routine lymph node dissection.

The following clinical and pathological variables were collected: age, comorbidities, parity, surgical procedure, stage according to the 2014 FIGO classification [35], final pathological analysis, adjuvant therapies, date of recurrence, death or latest news.

### 4.4. Sampling Protocol and Pathological Analysis

Peritoneal biopsies were taken at the end of the CRS by a senior surgeon experienced in ovarian cancer surgery. The end of the CRS was defined by complete removal of macroscopic PM. Sampling was performed according to the following protocol: surgeons took peritoneal biopsies of approximately 4 cm^2^. One biopsy was taken per location designated in the PCI if this location was initially affected by macroscopic PM (maximum of 13 biopsies per operation). Peritoneal biopsies were taken from areas outside of those that might suggest to the surgeon a scar of peritoneal metastases that had responded to neoadjuvant chemotherapy (NACT).

These samples were sent to the Pathology Department for analysis. Hematein, eosin, saffron (HES)-stained slides were read by both a specialized pathologist (JA) and a gynecologist (MC) to search for the presence of mPM. Microscopic PM were defined by the observation of adenocarcinoma foci on the HES slide of a biopsy sample or by the expression of PAX8 by isolated clusters of tumor cells by immunohistochemistry (IHC). The results were noted in the pathological report.

When a mPM was identified, tissue expression of *FRα* by immunohistochemistry was undertaken.

The Folate Receptor immunohistochemical study was performed on 4 µm thick sections of formalin-fixed, paraffin-embedded tissue using the mouse monoclonal antibody Folate Receptor Alpha (clone BN3.2, Leica Biosystems Newcastle Ltd., dilution 1/200). Tissue sections were placed on Super Frost Plus^®®^ glass slides and dried for 1 h in a dry oven at 58 °C. The technique was performed using the Ventana BenchMark Ultra automated system. Detection was performed using the UltraView Universal Dab Detection Kit using a Ventana CC1 solution at pH 6.0 for antigenic revelation. Sections of EOC overexpressing FRα were used as a positive external control. The IHC slides were all read in pairs by a specialized anatomopathologist and a gynecologist on a white light optical microscope.

For each slide, FRα expression was reported semi-quantitatively (0 = no labeling, 1 = low intensity, 2 = moderate intensity, 3 = high intensity), and the percentage of positive tumor cells was noted. A threshold of 5% was set to define the positivity of membrane and/or cytoplasmic expression of FRα on tumor cells, as described in several other studies.

### 4.5. Primary Endpoint

The primary endpoint was to assess FRα expression by mPM of HGSOC at the end of complete macroscopic CRS.

### 4.6. Statistical Analysis

Categorical variables were expressed as numbers (percentage). Continuous variables were expressed as means (+/- standard deviation, SD) or medians (range). Comparisons in patient characteristics between the two study groups (with mPM or without mPM) were performed using Student’s *t* test for continuous variables and the Mann-Whitney test for non-continuous variables. Statistical testing was performed at the two-tailed α level of 0.05. Data were managed with an Excel database (Microsoft Corporation, Redmond, WA, USA) and analyzed using R 3.5.1 software, available online.

### 4.7. Ethics

The research protocol was approved by a national Ethics Committee (PPC Sud Est II, ID-RCB 2017-03431-52; date of approval: 16 May 2018) and is registered on clinicaltrials.gov (ClinicalTrials.gov Identifier: NCT03754569). The collection of patient data was declared to the French “Commission Nationale Informatique et Liberté” (CNIL-Q9d2264273M). Each patient signed a written informed consent after receiving clear and detailed information about the research protocol.

## 5. Conclusions

Patients with advanced EOC present mPM after complete macroscopic CRS. FRα expression rate was found to be high among PM of patients with EOC peritoneal carcinomatosis. Targeting microscopic PM to eradicate any microscopic residual disease after conventional CRS may improve the high recurrence rate and patient survival. One promising approach would be the use of PDT by using photosensitizing molecules that specifically target FRα receptors overexpressed by EOC.

## Figures and Tables

**Table 1:** Population characteristics.

	Overall Population N = 26	without mPM N = 19	with mPM N = 7	*p*
Age (mean +/- SD)~years	65.3 +/- 11.1	63.6 +/- 11.6	69.9 +/- 9.1	0.17
BMI (mean +/- SD)~kg/m^2^	22.9 +/- 3.7	23.2 +/- 3.8	22.3 +/- 3.5	0.64
Body surface (mean +/- SD)~m^2^	1.65 +/- 0.13	1.65 +/- 0.15	1.62 +/- 0.04	0.91
Follow-up (after CRS), median [range]~days	492 [262–862]	495 [301–862]	376 [262–714]	0.27
Recurrence~n (%)	8 (30.8)	6 (31.6)	2 (28.6)	NA
RFS (median [range])~days	356 [213–862]	359 [292–862]	307 [213–400]	NA
Death~n (%)	3 (11.5)	2 (10.5)	1 (14.3)	NA
Peritoneal metastases spread at diagnosis				
- PCI (median [range])~/39	13 [3–31]	11 [3–31]	21.5 [10–31]	0.16
- Fagotti score (median [range])~/14	6 [0–12]	4 [0–10]	8 [6–12]	0.26
- CA 125 (median [range])~UI/mL	590 [19–8000]	579 [19–4042]	600 [40–8000]	0.57
Peritoneal metastases spread at CRS				
- PCI (median [range])~/39	11 [0–20]	11 [0–20]	4 [1–15]	0.40
- Fagotti score (median [range])~/14	4 [0–8]	4 [0–8]	2 [2–8]	0.97
- CA 125 (median [range])~UI/mL	20 [10–1162]	20 [10–1162]	253 [11–904]	0.30
FIGO Stage				
- IIIC~n (%)	18 (69.2)	13 (68.4)	5 (71.4)	NA
- IV~n (%)	8 (30.8)	6 (31.6)	2 (28.6)	NA
Number of biopsies (median [range])	7 [3–13]	7 [3–13]	6 [3–12]	0.41
NACT~n (%)	23 (88.5)	16 (84.2)	7 (100)	NA
Number of courses before CRS (median [range])	5 [3–9]	6 [3–9]	4 [3–9]	0.84

**Table 2:** FRα expression in patients with microscopic peritoneal metastases (percentage of FRα positive tumor cells; staining intensity).

	Diagnostic Biopsy (Exploratory Laparoscopy)	Macroscopic Peritoneal Metastases (Cytoreductive Surgery)	Microscopic Peritoneal Metastases (Cytoreductive Surgery)
1	(0%; 0)	(1%; 1) (5%; 2)	(0%; 0)
2	(5%; 1)	(0%; 0)	-
3	(20%; 2)	(50%; 2)	-
4	(0%; 0)	(60%; 3)	(20%; 1)
5	(60%; 2)	(30%; 3) (75%; 3)	(40%; 2)
6	(10%; 1)	(5%; 1) (0%; 0)	(25%; 1)
7	-	(5%; 1) (0%; 0)	-

**Table 3:** FRα expression in patients without microscopic peritoneal metastases (percentage of FRα positive tumor cells; staining intensity).

	Diagnostic Biopsy (Exploratory Laparoscopy)	Macroscopic Peritoneal Metastases (Cytoreductive Surgery)
1	(75%; 3)	(10%; 1)
2	(0%; 0)	(0%; 0)
3	-	(70%; 3)
4	-	(10%; 2)
5	-	(3%; 1)
6	-	(0%; 0)
7	-	(0%; 0)
8	(0%; 0)	(0%; 0)
9	-	(60%; 3)
10	-	(60%; 2)
11	-	(5%; 1)
12	-	(70%; 3)
13	(0%; 0)	(0%; 0) (15%; 1)
14	-	(40%; 1)
15	-	-
16	(30%; 2)	(0%; 0) (15%; 1)
17	(0%; 0)	(90%; 3)
18	-	(60%;2) (5%; 1)
19	(0%; 0)	(30%; 3) (5%; 1)

## Data Availability

The data presented in this study are available on request from the corresponding author.
